# The Syndrome of Elongated Styloid Process, the Eagle’s Syndrome—From Anatomical, Evolutionary and Embryological Backgrounds to 3D Printing and Personalized Surgery Planning. Report of Five Cases

**DOI:** 10.3390/medicina56090458

**Published:** 2020-09-09

**Authors:** Ladislav Czako, Kristian Simko, Andrej Thurzo, Branislav Galis, Ivan Varga

**Affiliations:** 1 Department of Oral and Maxillofacial Surgery, Faculty of Medicine, Comenius University in Bratislava and University Hospital, 81372 Bratislava, Slovakia; kristiansimko@gmail.com (K.S.); brano.galis@gmail.com (B.G.); 2 Department of Simulation and Virtual Medical Education, Faculty of Medicine, Comenius University in Bratislava, 81372 Bratislava, Slovakia; andrej@thurzo.sk; 3 Institute of Histology and Embryology, Faculty of Medicine, Comenius University in Bratislava, 81372 Bratislava, Slovakia; ivan.varga@fmed.uniba.sk

**Keywords:** elongated styloid process, Eagle’s syndrome, 3D printing, embryological development, evolutionary history, clinical anatomy

## Abstract

*Background and Objectives:* The symptoms of Eagle’s syndrome are associated with the elongated styloid process of the temporal bone or calcification of the stylohyoid ligament. The first mention of pain syndrome associated with the elongated styloid process dates back to 1937, when it was described by Watt Weems Eagle. Over the last decade, experts in the field have shown a lively interest in the issue of the relationship between the elongated styloid process and various symptoms. This article presents the correlation between the clinical signs of Eagle’s syndrome and alterations in surrounding anatomical structures. It includes a brief review of the evolutionary, embryological and clinical anatomical background of the elongated styloid process. *Materials and Methods:* Between 2018 and 2019, five patients were admitted to our workplace with 1–3-year history of bilateral or unilateral throat pain, otalgia and pharyngeal foreign body sensation. As a therapeutic novelty in the surgical approach to this condition, we used individual 3D printed models to measure and identify the exact location of the resection of the styloid process without damaging the surrounding anatomical structures, such as the facial, accessory, hypoglossal, and vagal nerves; the internal jugular vein; and the internal carotid artery. *Results:* Compared to traditional surgical methods without 3D models, 3D models helped to better identify cutting edges and major landmarks used in surgical treatment of Eagle’s syndrome. Printed models provided assistance with the exact location of the styloid process resection position without damaging the surrounding anatomical structures such as the facial, accessory, hypoglossal, and vagal nerves; the internal jugular vein; and the internal carotid artery. *Conclusion:* In our clinical report, we used 3D printed models for navigation and planning during surgical procedures involving resections of the elongated styloid process. Additionally, we can formulate a new hypothesis: the elongated styloid process is a form of atavism of the bony hyoid apparatus in our evolutionary ancestors that is evolutionarily encoded or arises from disrupted degeneration of the middle portion of embryonal Reichert´s cartilage of the second pharyngeal arch. Under normal conditions, this portion does not ossify but degenerates and transforms into a connective tissue band, the future stylohyoid ligament.

## 1. Introduction

Eagle’s syndrome is a condition involving the head and neck region, which is rarely identified anatomically and poorly understood clinically. The symptoms of Eagle’s syndrome are associated with the elongated styloid process of the temporal bone [1] or calcification of the stylohyoid ligament [2]. Considering a substantial variability in the length of styloid process in the general population, the styloid process should be considered “elongated” when longer than 3 cm [3]. In one reported case, the styloid process reached a length of 14 cm bilaterally [4]. Patients with Eagle´s syndrome typically present with a variety of symptoms, e.g., cervico-facial pain, foreign-body sensation in the throat, or dysphagia [5]. The first mention of pain syndrome associated with the elongated styloid process referred to as “stylalgia” dates back to 1937, when it was described by an American otorhinolaryngologist Watt Weems Eagle. Eagle and Durham [6] subsequently expanded on their initial descriptions, and reported that any styloid process longer than 2.5 cm could explain these symptoms. As in most eponymous cases, there are prior descriptions of stylohyoid elongation and stylohyoid ossification described by Marchetti in 1652 and Demanchetis in 1852, respectively. Eighteen years later in 1870, Lucke related this stylohyoid ligament calcification to painful syndrome [7]. In recent literature, Eagle’s syndrome has also been called stylohyoid syndrome, styloid syndrome, elongated process syndrome, stylalgia, styloid–stylohyoid syndrome, styloid dysphagia, chronic styloid angina, temporal rheumatic styloiditis, stylocarotid syndrome or Garel–Bernfeld syndrome [8].

**Figure 1:**
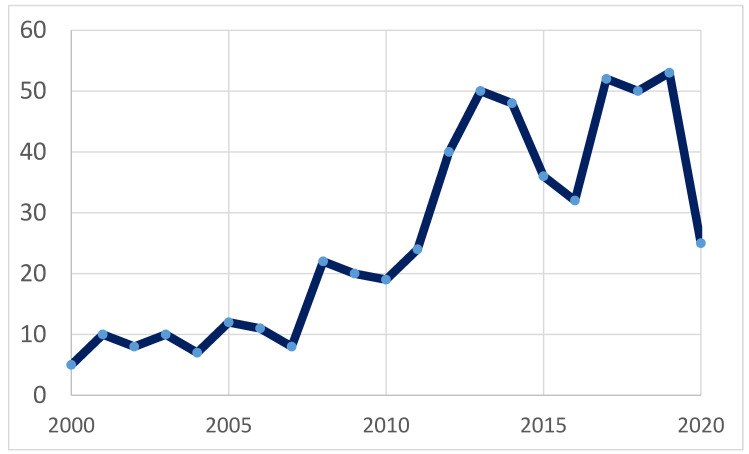
Number of scientific articles published per year (between 2000 and 2020) on the topic of Eagle´s syndrome or syndrome of elongated styloid process based on https://pubmed.ncbi.nlm.nih.gov/search results (National Center for Biotechnology Information, U.S. National Library of Medicine, on 7 June, 2020).

Over the last decade, experts in the field have shown a lively interest in the issue of the relationship between the elongated styloid process and various symptoms. The number of scientific papers concerning Eagle´s syndrome or the syndrome of the elongated styloid process published between 2000 and 2020 is summarized in Figure 1. According to the PubMed.ncbi.nlm.nih.gov database, more than 500 scientific papers have been published on the topic over the last 20 years, while the sharpest rise has been observed in the last decade. On the one hand, this phenomenon is surely brought about by enormous technological developments of medical imaging in the field of radiology. Nowadays, the diagnosis of the elongated styloid process is established by three-dimensional computed tomography [9] or cone beam computed tomography [10], which provide more precise topographic-anatomical and morphometric descriptions of this anatomical anomaly, compared to panoramic radiographs [11]. Considering the dynamic alteration of the anatomical relationship between the elongated styloid process and surrounding nerves and vessels relative to the head position, Siniscalchi [12] recommended that both magnetic resonance imaging (MRI) and ultrasonography of the head and neck region should be performed in different positions of the head (rest position, maximum extension, maximum flexion of the head, and maximum right and maximum left rotation). On the other hand, the interest in the issue of the elongated styloid process among surgical experts is growing due to expanding options of surgical approaches to the resection of this anatomical anomaly. To this day, no standardized treatment algorithm for the elongated styloid process has been established, although various surgical approaches have been described. Even though the traditional approaches (transcervical or transoral styloidectomy) are still in practice, novel modalities such as transoral robotic surgery have been employed lately in select patients, to avoid the potential shortcomings associated with other approaches [13]. Finally, yet importantly, it is necessary to emphasize perhaps the most significant factor in the “rise of popularity” of Eagle´s syndrome—a newly described neurological symptomatology possibly associated with the elongated styloid process. It can be a causative factor in the development of internal carotid artery compression [2], significant compression of the internal jugular vein [14,15], stylocarotid syndrome due to mechanical irritation of the sympathetic plexus in the cervical internal carotid artery [16] or stroke due to carotid artery dissection [17]. These recently described neurological morbidities possibly caused by the elongated styloid process have a tendency to spark the interest of many other fellow scientists, a phenomenon known as the “snowball effect”—one study builds upon the previous one. Therefore, the number of papers pointing to the importance of this anatomical variation in the pathogenesis of various conditions grows exponentially, which in turn leads to substantial attractiveness of the topic, yielding high numbers of citations. 

This article presents the correlation between the clinical signs of Eagle´s syndrome and alterations in surrounding anatomical structures. It includes a brief review of the embryological, phylogenetical, anatomical and clinico-anatomical backgrounds of the elongated styloid process and describes five cases of the elongated styloid process from our workplace, the Department of Oral and Maxillofacial Surgery, Faculty of Medicine, Comenius University in Bratislava, Slovakia. As a therapeutic novelty in the surgical approach to this condition, we used individual 3D printed models to measure and focus on identifying the exact location of the resection of the styloid process without damaging the surrounding anatomical structures, such as the facial, accessory, hypoglossal, and vagal nerves; the internal jugular vein; and the internal carotid artery.

## 2. Material and Methods

**Figure 2:**
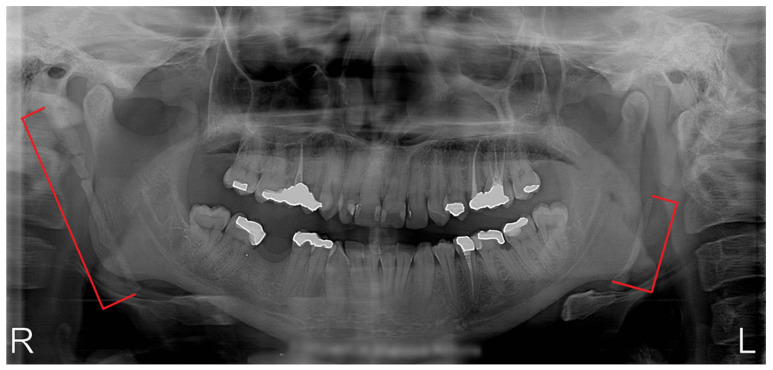
Panoramic radiograph of a patient with bilateral elongation of the styloid process.

**Figure 3:**
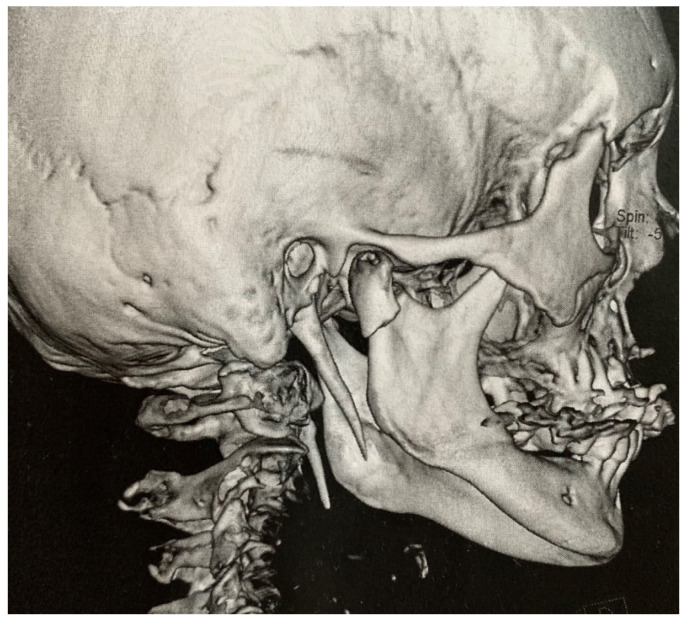
Three-dimensional computed tomography —an accidental finding of elongated styloid process in an asymptomatic patient with a fracture of the articular process of the mandible.

Between 2018 and 2019, five patients (age 44–77 year; 3 females and 2 males) were admitted to the Department of Oral and Maxillofacial Surgery, Faculty of Medicine, Comenius University in Bratislava, Slovakia, with 1–3-year history of bilateral or unilateral throat pain, otalgia and pharyngeal foreign body sensation. Two patients presented with dominant symptoms of dysphagia and pharyngeal foreign body sensation, one patient reported only bilateral neck pain with otalgia, one patient reported particularly unilateral neck pain accompanied by hypersalivation and voice change lasting for a few minutes after head rotation and one patient presented with symptoms of carotid compression—presyncope after rotating the head to the left. All patients had tried several courses of anti-inflammatory medication and multiple antibiotic therapies without sufficient effect. In all cases, a complete head and neck examination was performed (intraoral, extraoral); radiographic analysis (panoramic radiograph and computer tomography examinations, Figure 2 and Figure 3) of the head and neck region were obtained, and two patients underwent fiber optic laryngoscopy (due to foreign body sensation). Based on the clinical manifestations, radiological findings and digital palpation of the process in the tonsillar fossa, the diagnosis of Eagle´s syndrome was established. Four patients had bilateral elongation of the styloid process, and one patient was diagnosed unilaterally.

**Figure 4:**
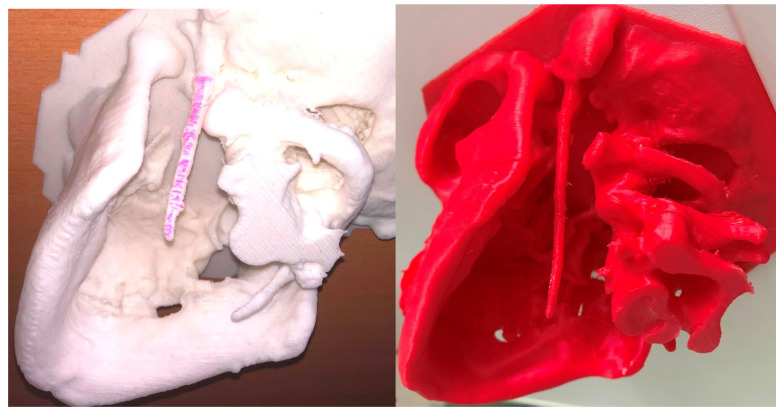
Two skull models fabricated by using three-dimensional printing technology reveal bilateral and unilateral elongated styloid processes.

During the surgery, we used individual 3D printed models to measure and focus on identifying the exact location of the resection of the styloid process without damaging the surrounding anatomical structures (Figure 4). A Form2 3D printer (Formlabs) was used to create all preoperative models. PreForm software was used to design 3D models of the temporal bone, mandible and other anatomical structures. To assess the fidelity of the 3D printed model, we inspected it from all perspectives. The model was examined to determine whether specific anatomical structures were located correctly and had the appropriate shape. All 3D printed models were scanned by computed tomography (CT) with the same parameters as those of the original temporal bone and mandible. The window level and window width of all images were set at 1000 and 4000 HU. The reproducibility of anatomical traits of given structures such as the length, angulation, form and position of the styloid process; the exact location of the stylomastoid foramen; the transverse process of the atlas; and the distance from the ascending ramus of the mandible were evaluated by comparing CT images of the 3D models to the original ones. Subsequently, we used this exact 3D model preoperatively, which helped to ensure better spatial navigation during the surgery and exact resection of the styloid process.

## 3. Results

**Figure 5:**
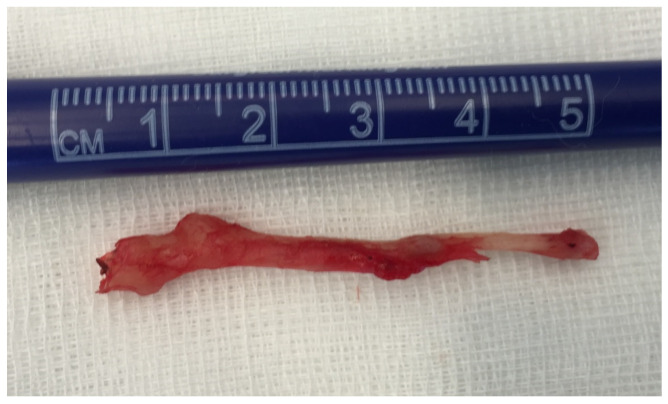
Resected elongated styloid process.

All five patients underwent resection of the styloid process from retromandibular approach; total of nine styloid processes were resected (length 4.8–7.2 cm; Figure 5). Postoperatively, four patients had immediate resolution of symptoms following their awakening in the recovery unit; one patient reported clinical improvement after 10 days. Patients were discharged from the hospital 3–5 days after the surgery, and had an uneventful post-operative course with minimal postoperative pain and swelling. Presently, 6 months after the surgery, all five patients are asymptomatic with minimal scarring and no peripheral neurological deficit.

## 4. Discussion

### 4.1. Clinico-Anatomical Remarks to Eagle’s Syndrome

The term ‘’styloid process’’ originates from the Greek “stylos”, which means a pillar. It gives attachment to numerous important ligaments (stylohyoid and stylomandibular) and muscles (styloglossal, stylohyoid and stylopharyngeus). The relationship between styloid process, surrounding blood vessels (internal jugular vein, external carotid artery, internal carotid artery and accompanying sympathetic chain), and cranial nerves (VII, IX, X, XI and XII) is important from the topographic point of view and is crucial to the ultimate pathophysiology of Eagle’s syndrome [4,8]. The elongated styloid process manifested as Eagle’s syndrome is characterized by a myriad of symptoms, which typically include pain in the anterolateral neck region, and in the area of the angle of the mandible, submandibular space and upper neck [6]. The typical clinical symptoms are recurrent or permanent throat pain, pharyngeal foreign body sensation, dysphagia, bilateral otalgia, recurrent neck pain and/or facial pain [18]. The pain is exacerbated by head rotation, lingual movements, speaking, swallowing, chewing, yawning and other oral and cervical movements. Eagle´s syndrome can be also accompanied by hypersalivation, and rarely by voice change lasting for a few minutes. All these symptoms are associated with an abnormal length, form and width of the styloid process [19]. 

There are several clinico-anatomical mechanisms that explain the exact cause of pain in patients with Eagle’s syndrome (Table 1). However, none of them could assess the development of all symptoms alone.

Incidence of abnormal stylohyoid length range from 4% to 28% [1,4]. The incidence is higher (up to 84%) if calcification of the stylohyoid complex is included [27]. Despite the fact that approximately 4% to 28% of the population is thought to have the elongated styloid process or calcification of the stylohyoid complex, only a small percentage (between 4% and 10.3%) is thought to be actually symptomatic [7,19]. To the best of our knowledge, no papers have dealt with the correlation between degree of the styloid process elongation and the severity of symptoms. 

From the anatomical point of view, there is an interesting correlation between the presence of elongated styloid process and a congenital anomaly of the first vertebra (atlas) known as *ponticulus posticus* [28]. *Ponticulus posticus* (or Kimmerle anomaly) is defined as an abnormal small bony bridge formed between the posterior portion of the superior articular process and the posterolateral portion of the superior margin of the posterior arch of the atlas, which encircles the vertebral artery [29]. The existence of these two congenital anomalies in one patient may be a mere coincidence or the result of simultaneous ossification of the stylohyoid ligament and posterior atlanto-occipital membrane during development [30].

Additionally, the elongation of styloid process is not the sole congenital anomaly of this bony projection of the temporal bone. Another congenital malformation is the presence of a sinus within the styloid process, termed “recessus processus styloidei” [31,32].

### 4.2. Evolutional and Embryological Background of Styloid Process Elongation

The hyoid apparatus in mammals provides the skeletal scaffolding supporting the tongue, upper vocal tract and larynx, and thus forms the core of the vocal production system. The hyoid apparatus of non-human mammals can vary in the extent to which its constituent parts are ossified, cartilaginous or ligamentous. This variation can occur among different species of the same order [33]. In humans, the styloid process, the stylohyoid ligament and the small horn of the hyoid bone form the hyoid apparatus. Several case reports have described completely ossified hyoid apparatus also in humans [34], even with diarthrodial—like joint formation [35,36]. We can hypothesize that the elongated styloid process in humans is evolutionary encoded and represents a form of atavism of the bony hyoid apparatus of our evolutionary ancestors.

From the embryonic perspective, the hyoid apparatus develops from the cartilage of Reichert of the second branchial (pharyngeal) arch. The further development of the Reichert´s cartilage in humans is not fully understood, as different scientists describe this process differently. During development, the Reichert’s cartilage produces five cartilaginous divisions (Table 2) [8,36,37,38,39]. These divisions are similar to bony elements of the hyoid apparatus of some tetrapod mammals [33]. In most cases, both tympanohyale and stylohyale undergo ossification to produce normal sized styloid processes. When only the tympanohyale ossifies, a short styloid process forms. In cases of extremely long styloid process or the complete ossification of hyoid apparatus, the ceratohyale does not degenerate, but undergoes ossification and completes the bony stylohyoid chain.

In contrast, Rodríguez-Vázquez et al. [40,41] described the further development of the Reichert’s cartilage in human embryos and fetuses in less complicated manner. The second branchial arch cartilage is formed in two independent segments (cranial—styloid and caudal—hyoid) separated by embryonic connective tissue—mesenchyme. The cranial segment is continuous with the otic capsule and becomes the styloid process. The caudal segment forms the majority of the hyoid bone. The intervening mesenchyme later disappears, and then probably gives rise to mentioned ligaments and muscles.

### 4.3. The Role of 3D Printing in Personalized Surgery Planning

3D printing techniques, also known as additive manufacturing, have been developing for the past three decades since 1986 (for review see [42]). However, the level of precision and accuracy required for clinical use was achieved only recently [43]. In combination with anatomically sensitive clinical imaging techniques such as CT or MRI scans, 3D printing technology in maxillofacial surgery offers important assistance in prosthetic rehabilitation, reconstruction and regeneration [44]. Low-cost 3D printing models have been used in medicine in many variations, including in preoperative planning, in surgical intraoperative guidance and in advancing medical education [42,45]. These synthetic models improve our understanding of preoperative anatomy in individual cases, allowing us to optimize our surgical approaches and introduce novel techniques tailored to a specific patient. This results in reduction of operative time, reduces surgical stress and minimizes perioperative errors [46,47]. In this clinical study, we reported five clinical cases assisted by 3D printing. We were able to achieve patient-directed customized anatomical approach and could identify major structural landmarks during preoperative planning. In addition, 3D printing technique enabled us to predict stability and prepare shapes of used alloplastic materials [44,47]. 3D printing models were also useful in understanding the relationship between the styloid process and surrounding key structures directly during the course of the surgery [48]. The basic step during the extraoral (submandibular, retromandibular) approach was the identification of the terminal part of the elongated styloid process. Based on this localization, the position of the styloid process was palpated. Preparation of the whole elongated pathological anatomical structure and complete resection subsequently followed. The surrounding anatomical structures (soft tissues) were identified during the preparation itself, while the perioperative assistance of the 3D model helped us to determine the extent and correct direction (depth) of the necessary preparation. Subsequently, it allowed us to directly perceive the surrounding anatomical structures and thereby prevent their damage by tissue preparation and retraction.

## 5. Conclusions

In our clinical report, we used 3D printed models for navigation and planning during surgical procedures involving resections of the elongated styloid process. Printed models provided assistance with the exact location of the styloid process resection position without damaging the surrounding anatomical structures. 3D printed models helped perioperatively to better identify cutting edges and major landmarks used in surgical treatment of the Eagle’s syndrome. In the future, in addition to 3D models, the use of virtual reality could also be considered. Virtual reality based on 3D CT examination could facilitate the process of preparation of the elongated styloid process with minimal risk of damage to the surrounding anatomical structures. The advantage of virtual reality could be in the preoperative simulation and practice of individual steps during surgery, consequently reducing the incidence of the most common perioperative complications of Eagle´s syndrome surgery (bleeding, nerve damage).

## Figures and Tables

**Table 1:** Accepted possible aetiologies of the Eagle’s syndrome and pathogenesis of clinical symptoms of the Eagle’s syndrome to date, reviewed in multiple papers [3,4,20,21,22,23,24,25,26].

1.	Mechanical Irritation of the Pharyngeal Mucosa from an Elongated Styloid Process
2.	Compression of nerves, including glossopharyngeal nerve, lower branch of trigeminal nerve, and/or the chorda tympani
3.	Fracture of ossified stylohyoid ligament by a sudden head movement, followed by growth of granulation tissue
4.	Pressure on the carotid artery that may affect the circulation and produce irritation of the sympathetic nerves of the arterial sheath
5.	Degenerative changes in the tendon of the stylohyoid muscle insertion
6.	Stretching and fibrosis involving the V, VII, IX, and X cranial nerves after tonsillectomy
7.	Traumas in the cervicopharyngeal region (as a possible result of “reactive metaplasia”)
8.	Developmental aspect—trauma during development of styloid process
9.	Psychological aspect—patient is more sensitive to the symptoms of elongated styloid process after a trauma or surgery
10.	Endocrine changes at menopause or increased serum calcium concentration and increased heel bone density
11.	Genetic aetiology—an autosomal dominant inheritance pattern

**Table 2:** Development of the hyoid apparatus in humans.

Divisions of the Embryonal Reichert´s Cartilage, from Cranial to Caudal Side	Derivatives in Adulthood
Tympanohyale	Styloid process (proximal part)
Stylohyale	Styloid process (distal part)
Ceratohyale	Stylohyoid ligament (partially possible participation in styloid process formation)
Hypohyale	Small horn of the hyoid bone
Basihyale	Upper body of the hyoid bone
